# Medical Missions

**Published:** 1913-04-05

**Authors:** 


### MEDICAL MISSIONS.

The recent celebration of the centenary of David Livingstone's birth has served to direct attention to the whole subject of medical missions and their aims. Sometimes, as in Livingstone's case, an earnest missionary takes a medical degree as part of his training for the better conversion of the heathen to Christianity. More often, a doctor, who has deep religious convictions, decides to make some far country the scene of his professional labours; conversions being in this case the second rather than the chief object of his life's work. In any case there can be few who will deny that a successful medical mission—and medical missions, we are glad to say, are very seldom failures—is a real blessing to the peoples, be they Negroes, Mongolians, or what not, amongst whom it is planted. Introduced to the benefits of Western medicine and surgery, they realise tangibly enough that they are getting something far better than they have ever had before; and they get also an admirable example of the ethical spirit of Christianity, which is bound to dispose them favourably to those who expound its teachings.

Without any disrespect to the great explorer who was born a hundred years ago, we may doubt whether his professional equipment was a twentieth as efficient as that of many of the medical missionaries of to-day. The whole of antiseptic surgery, for instance, must have been a sealed book to him; not, of course, through any fault of his own. But nowadays the typical medical missionary is a person whose capacity for general and special practice must be great indeed. The Baghdad Medical Mission, for instance, gets through four or five hundred major operations a year; and is extending itself into new premises on a new site where three or four times this amount of work will be undertaken. Nor is this an exceptional case; everywhere the medical missions are finding themselves with overflowing clinics, especially in surgery, and are working at a pressure which is rapidly getting higher. One result of this is bound to be the differentiation of the medical man and the proselytising missionary. The former gets no time for preaching; the latter finds that a smattering of medical knowledge is no longer enough to work wonders with. In other words, the Livingstone type is no longer likely to achieve the successes of last century. Rather does the future of medical missions lie in the hands of such men as Pennell, Gibb, and Pain, three who have succumbed to disease during the past year in different quarters of the globe. The former was the man of whom it was said that his influence with the Pathans was worth two regiments of soldiers. We mention these three because they are no longer with us; but there are scores of others doing work as good as theirs was, and worthily upholding the credit of British medicine in many countries.

There is another fact about missionary work which has been too much overlooked, and that is that the missionary, more than any other protagonist of the faith, has to meet practical rather than theological difficulties. It has been well said that if you want to appeal to the spirit of your fellows you must get at them through their religion rather than through your own; and the religion of the East and those peoples to whom foreign missions are sent may be described broadly as a religion of customs. That at any rate is the expression of the religions which most impresses the missionary, but it is doubtful whether a training in theology is itself sufficient to enable him to approach these customs with sympathy. Without sympathy he will do nothing, and in order to obtain it science and medicine must also have a place with theology in missionary training. That is why the medical missions have been the most successful, and why they have most deserved success.

